# Chemometric Simultaneous Estimation of Clopidogrel Bisulphate and Aspirin from Combined Dosage Form

**DOI:** 10.4103/0250-474X.44592

**Published:** 2008

**Authors:** S. J. Rajput, R. K. George, Deepti B. Ruikar

**Affiliations:** Pharmaceutical Quality Assurance Laboratory, Pharmacy Department, Faculty of Technology and Engineering, The M. S. University of Baroda, Vadodara-390 001, India; 1 Applied Mathematics Department, Faculty of Technology and Engineering, The M. S. University of Baroda, Vadodara-390 001, India

**Keywords:** Chemometrics, clopidogrel bisulphate, aspirin

## Abstract

Two chemometric methods, inverse least square and classical least square, were applied to simultaneous assay of clopidogrel bisulphate and aspirin in their combined dosage tablet formulation. Twelve mixed solutions were prepared for the chemometric calibration as training set and 10 mixed solutions were prepared as validation set. The absorbance data matrix was obtained by measuring the absorbance at 16 wavelength points, from 220 to 250 nm with the interval of 2 nm (Δλ= 2 nm). The developed calibrations were successfully tested for laboratory mixtures as well as commercial tablet formulation for their clopidogrel bisulphate and aspirin concentration. Mean recoveries for clopidogrel bisulphate and aspirin were found to be in good agreement with the label claim.

Clopidogrel bisulphate (CLP), [S-(a)(2-chlorophenyl)-6,7-dihydrothieno (3,2-C)-pyridine-5(4H) acetic acid methyl ester sulphate]^1^ and aspirin (ASP), acetylsalicylic acid^2^ are used as antiplatelet agents in their combined dosage formulations. The tablet formulation is a somewhat new entrant in the Indian market. Aspirin is a well studied drug, official in all the pharmacopoeias whereas clopidogrel is not official in any of these pharmacopoeias^3^–^5^. Several spectrophotometric^6^ and HPLC^7^–^10^ methods are reported for the estimation of aspirin in literature, whereas only a few HPLC^11^–^13^ methods are available for clopidogrel bisulphate. A spectrophotometric method^14^ was reported recently in literature for simultaneously analyzing ASP and CLP where the analysis was done after hydrolyzing the drugs.

In recent years, the multivariate or chemometric methods such as classical least square (CLS), inverse least square (ILS), principal component regression (PCR) and partial least square (PLS), based on computer assisted instrumentation have been applied for the analysis of multicomponent analysis of mixtures^15^–^17^. The application of multivariate calibration to the absorbance signals produced by drugs during their simultaneous determination in pharmaceutical preparations is an effective means for their quality control. These methods were found to be well suited to analyze the drugs with highly overlapped spectra. The aim of the present study was to investigate the ability of ILS and CLS methods to quantify the two component mixture of CLP and ASP with overlapping spectra. The proposed methods are simple and accurate and resulted in significant reduction in analysis time.

In the present investigation, two chemometric methods based on ILS and CLS are successfully applied to the simultaneous determination of CLP and ASP in a commercial tablet formulation without any separation procedure. The chemometric calibrations were carried out by using the standard mixtures of these drugs in various compositions and concentrations. The methods were validated after establishing mean recoveries and relative standard deviations of both the methods. Amount of dissolved drugs in laboratory samples and commercial tablet formulation were calculated by the chemometric methods. The results obtained were compared with each other.

## MATERIALS AND METHODS

Aspirin and clopidogrel bisulphate were obtained as gift samples from Zydus Cadila, Ahmedabad. AR grade methanol from Qualigens, Mumbai, was used as a solvent for preparing stock solutions of CLP and ASP (100 μg/ml). Commercially available Deplatt A-150 (Torrent Pharmaceuticals), containing 75 mg of CLP and 150 mg of ASP per tablet, was randomly selected for the study and the tablets were procured from the local market.

Shimadzu UV-1701 double beam UV/Vis spectrophotometer, loaded with UVPC software was used for spectral measurements. Additional softwares MATLAB 6.1 and EXCEL, were used for the computation of spectral data, chemometric calculations and statistical analysis.

### Experimental design:

Chemometric method or multivariate analysis was carried out using ILS and CLS methods. The methods are carried out in two steps. In the first step, an empirical mathematical model was built representing the relationship between the absorbance and concentration data generated from a set of standard samples (calibration set). The second step is the prediction step in which the calibration model is used to determine the concentration of the components (validation set) from their spectral data. The accuracy and precision of predictive ability of the model can be defined as root mean square error of prediction (RMSEP). A mathematical model is accepted if the value of RMSEP units is found to be less than three. Inverse least square (ILS) is the application of multiple linear regression (MLR) to the inverse expression of the Beer-Lambert’s law of spectroscopy. The mathematical expression is given as, C= P×A.. (1), where C is the concentration matrix, A is the absorbance matrix and P is the calibration constant. The above equation can be written as a linear equation system as follows, C_1_ = P_11_ .A_1_ +P_12_ .A_1_ +....P_1w_ +A_w_, C_2_ = P_21_ .A_1_ +P_22_ .A_2_ +....P_2w_ +A_w_ or C_c_ = P_c1_ .A_1_ +P_c2_ .A_2_ +....P_cw_ +A_w_, where A_w_ is the absorbance at w^th^ wavelength, P_cw_ is the calibration coefficient for the c^th^ component at w^th^ wavelength and C_w_ is the concentration of c^th^ component.

The classical least square (CLS) calibration technique, based on linear CLS algorithm, involves the calculation of K, calibration coefficient using the absorbance data from the calibration set. The linear CLS algorithm has the following steps, A=K×C…(2). In this equation, A is the absorbance matrix, C is the concentration matrix and K is the calibration constant. The linear form of the equation can be written as, A_1_ = K_11_ .A_1_ +K_12_ .C_2_ +....K_1c_ +C_c_, A_2_ = K_21_ .A_1_ +K_22_ .C_2_ +....K_2c_ +C_c_, Or A_w_ = K_w1_ .A_1_ + K_w2_ .C_2_ +.... K_cw_ +C_c_, where A_w_ is the absorbance at w^th^ wavelength, K_cw_ is the calibration coefficient for c^th^ component at w^th^ wavelength and C_c_ is the concentration of c^th^ component. The calibration Eqns 1 and 2 are used for the estimation of the amounts of components in the samples.

### Preparation of solutions:

A training set (calibration set) was prepared containing 0-30 μg/ml of CLP and 0-20 μg/ml of ASP in twelve varied compositions (Table 1). A validation set containing 10 synthetic mixtures of CLP and ASP solutions in various possible combinations (Table 2) was prepared from the stock solution.

**TABLE 1:** COMPOSITION OF CALIBRATION SET FOR ILS AND CLS METHODS

Mixture	Aspirin concentration (μg/ml)	Clopidogrel bisulphate concentration (μg/ml)
1	4	10
2	8	10
3	10	10
4	16	10
5	20	10
6	12	0
7	16	6
8	16	12
9	16	18
10	16	24
11	16	30
12	0	10

The mixture composition was selected randomly to have varied proportions of ASP and CLP.

**TABLE 2:** COMPOSITION OF VALIDATION SET FOR ILS AND CLS METHODS

Mixture	Aspirin concentration (μg/ml)	Clopidogrel bisulphate concentration (μg/ml)
1.	12	9
2.	14	9
3	16	9
4	18	9
5	20	9
6	18	6
7	18	9
8	18	12
9	18	15
10	18	18

All possible proportions were included in selecting the composition of validation set.

### Analysis of tablet formulations:

Twenty tablets were taken and their average weight was determined. The tablets were then triturated to a fine powder and the powder equivalent to 50 mg CLP was taken in a 50 ml volumetric flask. The powder was dissolved in methanol by intermittent shaking for 4-5 min. The volume was made upto 50 ml with methanol and the solution was filtered through Whatman filter paper (no. 41). The filtrate was further diluted with methanol to get the sample solutions.

### Recovery studies:

The recovery studies were performed by spiking a preanalyzed tablet solution with definite and different concentrations of pure drug. The experiment was repeated six times to emphasize the accuracy.

### Statistical analysis:

The ability and efficiency of calibration was studied by estimating the standard variation of chemometric calibrations in case of investigated mixtures. The root mean square error of prediction (RMSEP) was calculated from the following formula, 
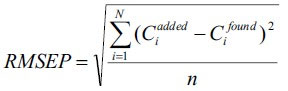


where, C_i_^added^ = added concentration of the drug and Ci^found^ = predicted concentration of the drug. One way ANOVA was also applied to compare the methods.

## RESULTS AND DISCUSSION

**Fig. 1:**
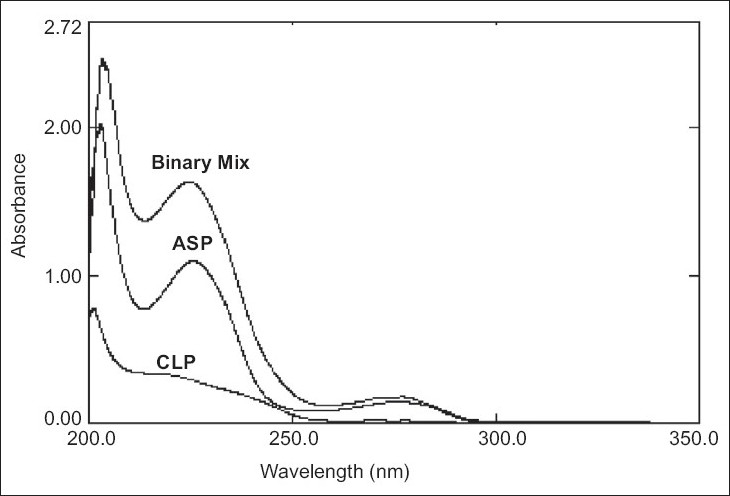
Overlain spectra of CLP, ASP and their mixture

The zero order UV spectra (fig. 1) of CLP and ASP completely overlap with each other making it impossible to use the conventional spectrophotometric methods to analyze CLP and ASP, in presence of each other. Mathematical models based on multivariate calibration were then applied to analyze these drugs.

The calibration set was randomly prepared with the mixtures of CLP and ASP in methanol (Table 1). The UV spectra of this calibration set were recorded in the spectral region between 200 - 310 nm. The absorbance were measured at 16 wavelength points from 220 to 250 nm with the interval of 2 nm (y=2 nm). The spectra were stored using UVPC software. The chemometric calibration was computed with the ILS and CLS algorithms using the correlation for the training set and the absorbance data. The contents of CLP and ASP in the laboratory mixtures and tablets were calculated by chemometric calculations. Mean recoveries, relative standard deviation and other statistical parameters of ILS and CLS were calculated. The results are shown in Table 3-5 respectively.

**TABLE 3:** ANALYSIS OF VALIDATION SET BY ILS METHOD

Mixture	Aspirin			Clopidogrel bisulphate		
		
	Amount taken (μg/ml)	Amount found (μg/ml)	% recovery	Amount taken (μg/ml)	Amount found (μg/ml)	% recovery
1.	12	11.45	95.41	9	8.85	98.33
2.	14	13.69	97.78	9	9.30	103.33
3.	16	15.82	98.87	9	8.93	99.22
4.	18	18.10	100.55	9	9.01	101.11
5.	20	20.60	103	9	8.76	97.33
6.	18	17.80	98.88	6	5.88	98
7.	18	17.65	98.05	9	9.07	100.77
8.	18	17.63	97.66	12	11.89	99.08
9.	18	17.78	97.94	15	15.08	100.53
10.	18	17.58	98.69	18	17.59	97.72

Mean recoveries,%RSD and RMSEP for ASP and CLP were 98.69 and 99.45, 1.91 and 1.75 and 0.236 and 0.19, respectively.

**TABLE 4:** ANALYSIS OF VALIDATION SET BY ILS METHOD

Mixture	Aspirin			Clopidogrel bisulphate		
		
	Amount taken (μg/ml)	Amount found (μg/ml)	% recovery	Amount taken (μg/ml)	Amount found (μg/ml)	% recovery
1	12	12.10	100.83	9	8.95	99.44
2.	14	13.98	99.85	9	9.15	101.66
3.	16	15.85	99.06	9	9.10	101.11
4.	18	18.15	100.83	9	8.76	97.33
5.	20	19.81	99.05	9	8.60	95.55
6.	18	18.08	100.44	6	6.21	103.50
7.	18	17.65	98.05	9	9.18	102.0
8.	18	17.91	99.50	12	11.81	98.41
9.	18	18.10	100.55	15	15.15	101.0
10.	18	18.20	101.11	18	18.15	100.83

Mean recoveries,%RSD and RMSEP for ASP and CLP were 99.92 and 100.8, 0.944 and 1.570 and 0.161 and 0.19, respectively.

**TABLE 5:** ANALYSIS OF TABLET FORMULATION

Formulation	Label claim, mg/tab	ILS mg/tab found	CLS mg/tab found
Deplatt A-150	CLP 75	74.45 ± 0.69	74.66 ± 1.17
	ASP 150	148.92 ± 1.53	150.85 ± 0.34

The results shown are the mean values of six determinations.

In the ILS method, the coefficient matrix (P) was obtained from the linear equation system using the absorbance data and the training set. Introducing (P) into the linear equation, the calibration matrix for ILS was obtained. In the CLS method, the corresponding calibration coefficient (K) was obtained using CLS algorithm.

To select the number of factors in algorithms, a cross validation method leaving out one sample at a time^18^ was employed. Using the calibration set spectra, the concentration of the sample left out was predicted. The predicted concentrations of the components in each sample were compared with the actual concentration and RMSEP was calculated. The RMSEP was used as a diagnostic test for examining the errors in the predicted concentrations. It indicates the precision and accuracy of predictions.

The ILS and CLS methods were applied to the determination of synthetic mixtures containing various concentrations of CLP and ASP to check the validity of the calibration models. These calibration models were used to predict the concentrations of the drugs in 10 synthetic mixtures. The tested mixtures were subjected to recovery studies. The results obtained were quite satisfactory. These results, mean recoveries and relative standard deviation are shown in Table 3 and 4. The models were then applied to estimate CLP and ASP in tablets. The results are shown in Table 5. The numerical values of all statistical parameters indicated that the proposed techniques are suitable for the determination of these drugs in the tablet formulation as excipients do not interfere.

One way ANOVA test was applied to four sets of ten samples for each drug in tablet formulation for comparing the difference between the ILS and CLS methods. For this, Snedecor’s F-values were computed and compared with the standard tabulated values (P=0.05). The same computation process was repeated for the drugs. In standard table, for n_1_ = 2 and n_2_ = 27 (P=0.005), the F-value was 3.35. ANOVA test results were calculated as 2.39 for CLP and 1.18 for ASP (Table 6). The experimental (calculated) F-values did not exceed the F-tabulated values indicating no significant difference between the methods.

**TABLE 6:** DATA FOR PRECISION STUDY USING ONE WAY ANOVA

Parameter	ILS		CLS	
		
	CLP	ASP	CLP	ASP
Between day variance	5.30	3.02	10.20	4.12
Within day variance	3.46	2.09	1.39	2.61
F-ratio	2.35	1.32	0.58	2.54
TSS	168.16	89.70	287.44	132.84

One way ANOVA test was applied to four sets of ten samples. TSS is the total sum of squares.
